# Prostate brachytherapy followed by secondary urethral adenocarcinoma: A case report

**DOI:** 10.1016/j.eucr.2026.103495

**Published:** 2026-05-29

**Authors:** Daniel M. Callahan, Hayes W. Miller, Ryan L. Frazier, E. Charles Osterberg, Aaron A. Laviana

**Affiliations:** a Dell Medical School at the University of Texas at Austin, USA; b Department of Surgery and Perioperative Care, Dell Medical School, USA; c Urology Austin, USA

**Keywords:** Prostate brachytherapy, Secondary malignancy, Urethral adenocarcinoma, Radiation-induced cancer, Urethral stricture, HER2 amplification

## Abstract

Prostate brachytherapy carries a risk of secondary malignancies, with data regarding secondary urethral malignancies being particularly sparse. We report a case of secondary urethral adenocarcinoma presenting 14 years after low-dose-rate (LDR) brachytherapy.

A 57-year-old man developed recurrent urethral stenosis after LDR brachytherapy for prostate cancer (2009). In 2023, subsequent resections confirmed HER2-positive, ERBB2-amplified urethral adenocarcinoma. After neoadjuvant oxaliplatin/capecitabine (CAPOX) therapy, he underwent cystoprostatectomy, urethrectomy, and ileal conduit. Pathology showed stage IIIB disease with negative margins.

This case underscores a rare but severe complication of prostate brachytherapy, contributing to the clinical understanding of secondary urethral malignancies.

## Highlights

• First report of urethral adenocarcinoma 14 years post-brachytherapy.
• Chronic urethral stricture may signal malignant transformation risk.
• HER2 amplification identified, supporting targeted therapy approach.
• ctDNA aided staging and supported localized disease assessment.
• Multimodal surgery achieved negative margins in advanced disease.

## 1 Introduction

Prostate cancer is the most prevalent non-cutaneous malignancy among men in the United States and the second most common worldwide.^1^ In 2025, an estimated 313,780 new cases and 35,770 deaths were projected in the U.S. alone, following a global burden of 1.47 million cases in 2022.^1^, ^2^, ^3^ While active surveillance remains the preferred strategy for low-risk and select favorable intermediate-risk disease, curative-intent local therapy—including radical prostatectomy, external beam radiotherapy (EBRT), and brachytherapy (BT)—is indicated for patients with intermediate-to high-risk localized disease and sufficient life expectancy.^4^

EBRT and BT offer comparable tumor control rates but differ significantly in delivery and temporal impact. EBRT utilizes precisely targeted, high-energy X-ray fractions over several weeks.^5^ Conversely, BT involves the placement of radioactive sources directly within or adjacent to the tumor. This is achieved via high-dose-rate (HDR) temporary catheterization or low-dose-rate (LDR) permanent seed implantation.^6^ While EBRT is associated with higher long-term gastrointestinal (GI) toxicity, BT is more frequently linked to acute and chronic genitourinary (GU) complications, most notably urethral stenosis.^7^

The side-effect profiles of these modalities often overlap and may be exacerbated when used synergistically. At five years post-treatment, common sequelae include urinary irritative symptoms (15.8% for LDR brachytherapy boost vs. 10.4% for EBRT monotherapy) and bowel dysfunction (32.3% vs. 18.7%).^8^ Rare but severe complications include fistula formation and secondary pelvic malignancies.

LDR BT, characterized by prolonged radiation exposure and potential “hot spots” of chronic inflammation from sporadic seeding, has been specifically implicated in an increased risk of secondary GU malignancies compared to radical prostatectomy (6.0% vs. 2.4%, HR 1.58).^9^ Recent longitudinal studies report the incidence of secondary pelvic malignancies with LDR at 1%–2% at 10 years, rising to 6.4% and 9.8% at 15 and 20 years, respectively.^10^ This risk is multifactorial, modulated by patient-specific variables (age, smoking history, genetic predisposition) and treatment parameters (fractionation, irradiated volume, and latency).

Although the literature on secondary bladder malignancy after brachytherapy is plentiful, secondary urethral malignancies represent an exceedingly rare clinical entity, with limited data to guide management. This report describes a patient who developed secondary urethral adenocarcinoma following a significant latency period after brachytherapy for primary prostate adenocarcinoma.

## 2 Case presentation

In 2009, a 57-year-old male patient with a known Factor V Leiden mutation was diagnosed with Grade Group 2 prostate adenocarcinoma. He underwent definitive low-dose-rate (LDR) brachytherapy monotherapy. His post-treatment course remained unremarkable until seven years post-implantation, when he presented with progressive obstructive lower urinary tract symptoms (LUTS).

Diagnostic workup revealed a posterior prostate-membranous urethral stricture. The patient was initially managed with recurrent urethral dilations for several years. He subsequently underwent drug-coated balloon dilation (Optilume®) in 2022 for a 1 cm refractory segment. Despite these interventions, the stricture remained recurrent. Throughout this time frame, his serum prostate-specific antigen (PSA) levels remained nearly undetectable (highest recorded value 0.042).

In 2023, the patient underwent posterior urethroplasty, brachytherapy seed removal, and subtotal prostatectomy for refractory stenosis. Histopathological analysis of the resected tissue demonstrated small foci of adenocarcinoma with signet ring features. These findings were characterized as a post-radiation adenocarcinoma of urothelial/urethral origin, distinct from his primary prostatic malignancy. Following excision with negative margins, serial PSAs were persistently at subclinical values (never reaching above 0.03). Therefore, adjuvant therapy was deferred due to localized disease and low follow-up PSAs.

In January 2025, the patient presented with gross hematuria and acute urinary retention. Cystoscopic evaluation identified a *de novo* lesion in the prostatic urethra at the prior anastomotic site, suspicious for a secondary malignancy. Transurethral resection confirmed a moderately differentiated, enteric-type adenocarcinoma with fibromuscular involvement. Molecular profiling revealed HER2 IHC 3+ expression, *ERBB2* amplification, and MSI stability. A subsequent colonoscopy was negative for primary bowel malignancy, further supporting a primary urinary tract origin.

Restaging in March 2025 via repeat transurethral resection confirmed persistent tumor within the membranous urethra, prostatic urethra, and bladder neck, encompassing the prior anastomotic site. F-18 fluorodeoxyglucose positron emission tomography (FDG-PET) imaging demonstrated localized hypermetabolic activity in the left prostatic lobe and urethra, without evidence of distant metastatic disease. Circulating tumor DNA (ctDNA) testing was negative.

Based on the extensive posterior urethral involvement, the patient initiated neoadjuvant chemotherapy with three cycles of capecitabine and oxaliplatin (CAPOX). This was chosen to due urethral adenocarcinoma's histologic behavior which is similar to colorectal cancers, for which CAPOX is a standard of care.^11^^,^^12^ In September 2025, he underwent definitive robotic-assisted radical cystoprostatectomy, total urethrectomy, bilateral pelvic lymphadenectomy, and an omental pedicle flap with ileal conduit urinary diversion.

**Fig. 1a-d:**
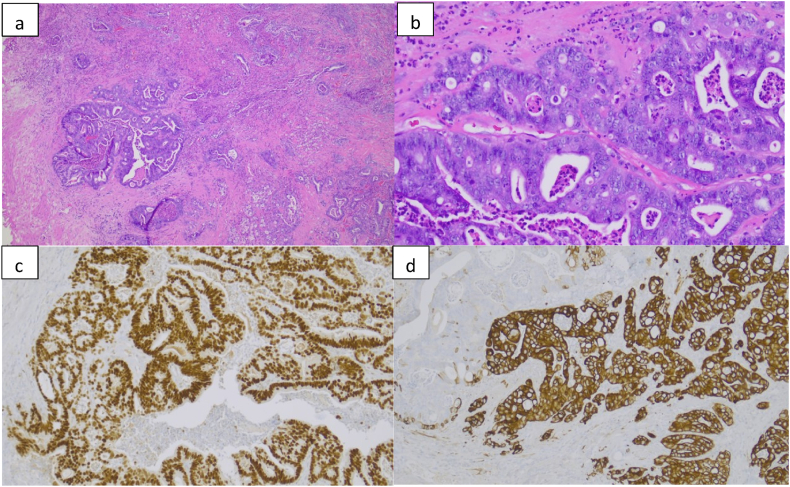
Histopathologic and immunohistochemical features of tumor. (a) Low-power hematoxylin and eosin (H&E) stain illustrating infiltrative glandular neoplasm with irregular glands within a desmoplastic stroma. (b) High-power H&E stain showing complex glandular architecture with cytologic atypia, hyperchromatic nuclei, prominent nucleoli, and luminal mucin production. (c) Nuclear immunohistochemical (IHC) staining showing diffuse nuclear positivity in tumor cells. (d) Cytoplasmic/membranous IHC staining showing epithelial differentiation with adjacent non-neoplastic tissue.

Final surgical pathology revealed remaining Grade Group 4 (Gleason Score 4 + 4 = 8) prostate adenocarcinoma at the bladder neck, distinct from the urethral cancer, with negative surgical margins and negative lymph nodes. The final urethral pathology showed mucosal ulceration in a background of chronic urethritis and granulation tissue reaction no malignancy or dysplasia identified, supporting resolution of urethral disease after neoadjuvant chemotherapy. Pathology is shown in Fig. 1a–d. At the six-month follow-up, the patient remains clinically free of disease with a ctDNA score of zero.

## 3 Discussion

In this report, we describe a rare case of secondary urethral adenocarcinoma presenting 14 years after low-dose-rate (LDR) brachytherapy (BT) for primary prostate cancer. While prostate BT is an established curative modality for localized disease, its long-term sequelae—specifically secondary malignancies—warrant heightened clinical attention due to the extended life expectancy of this patient population.^7^^,^^13^

The biologic plausibility of late radiation-associated secondary tumors is well-supported. Hoffman et al. previously demonstrated that patients undergoing radiation often differ demographically from those undergoing surgery, typically presenting with more comorbidities.^14^ Furthermore, a systematic review and meta-analysis by Wallis et al. identified a significantly increased risk of secondary bladder and rectal malignancies following radiotherapy compared to radical prostatectomy, with risk profiles escalating beyond a 10-year follow-up period.^15^ Consequently, comprehensive counseling regarding long-term oncologic and functional outcomes is essential for facilitating preference-concordant treatment decisions.

This case underscores that late-onset urinary symptoms following prostate radiotherapy should not be reflexively dismissed as benign radiation toxicity. The clinical course illustrated here highlights the diagnostic complexity inherent in post-radiation malignancies, particularly at atypical sites such as the urethra.

Initially, tissue obtained during urethroplasty and subtotal prostatectomy raised suspicion regarding the malignancy's primary origin. This diagnostic ambiguity necessitates a multidisciplinary approach and expert uropathological review. Distinguishing between recurrent prostate adenocarcinoma, *de novo* urothelial carcinoma with glandular features, and enteric-type adenocarcinoma of the lower urinary tract requires the careful integration of morphological assessment, immunohistochemical (IHC) profiling, and increasingly, tumor genomic sequencing.^16^^,^^17^ Precise characterization has profound practical implications: it dictates whether PSA-based monitoring is clinically relevant, determines whether the treatment should mirror urothelial or enteric paradigms, and assists in the interpretation of focal advanced imaging (e.g., PET-avidity) when conventional cross-sectional imaging remains equivocal.

While secondary urethral malignancies following prostate brachytherapy (BT) are historically associated with advanced stage and poor prognosis, the present case was notable for localized disease only. This clinical suspicion was corroborated by novel diagnostic adjuncts, including circulating tumor DNA (ctDNA) monitoring. Our strategy aligns with data from Patel et al., who reported a 98.5% concordance between ctDNA detection and primary urethral cancer status.^18^ Although ctDNA sensitivity is not absolute, an undetectable ctDNA level in the context of negative high-resolution staging imaging provided critical reassurance for proceeding with aggressive extirpative surgery.

Furthermore, the identification of *ERBB2* amplification and HER2 overexpression in this case provided a clear rationale for targeted systemic therapy. This underscores the expanding role of molecular profiling in post-radiation urethral tumors, where histologic subtype alone may be insufficient to guide precision oncology. To our knowledge, the integration of HER2-directed therapy for this specific post-radiation entity has not been previously described.

A defining feature of this case was the patient's protracted history of recurrent prostate-membranous urethral stenosis. Urethral stricture disease is a recognized late sequela of radiotherapy; literature on high-dose-rate (HDR) BT suggests that nearly 50% of affected patients require multiple interventions for recurrence.^19^

We hypothesize a synergistic pathway for carcinogenesis in this setting: initial radiation-induced injury triggers chronic ischemia and fibrosis, while subsequent repeated instrumentation and persistent inflammation facilitate epithelial remodeling, squamous metaplasia, and dysplasia. These chronic inflammatory states create a permissive microenvironment for malignant transformation. This association is bolstered by prior reports of urethral carcinoma arising after complex urethroplasty.^20^ Clinicians should maintain a low threshold for biopsy in patients with recurrent stenosis following pelvic brachytherapy—particularly when presenting with atypical hematuria or rapid recurrence—rather than relying solely on repeated empiric dilation.

As the population of prostate cancer survivors continues to expand, the incidence of rare but highly aggressive secondary pelvic malignancies is expected to rise. This case contributes to the sparse literature characterizing secondary urethral and bladder neck malignancies following definitive brachytherapy. Our findings underscore the critical necessity for maintaining a high index of clinical suspicion when managing recurrent or refractory urethral stricture disease in the post-radiation setting. Awareness of these late-onset sequelae is paramount to ensuring timely diagnosis, facilitating comprehensive molecular characterization, and implementing definitive, life-saving therapeutic interventions.

## 4 Conclusion

While brachytherapy remains a cornerstone of curative-intent treatment for prostate cancer, this case highlights the critical need for clinical vigilance regarding secondary malignancies in patients presenting with subsequent urethral stricture disease. Although radiation-induced urethral adenocarcinomas often exhibit an aggressive phenotype and present at advanced stages, prompt diagnostic biopsy and early identification are essential to facilitate radical extirpation and potentially improve long-term oncologic outcomes. This case emphasizes that new-onset or refractory lower urinary tract symptoms in the post-radiation setting must be investigated beyond benign obstructive etiologies.

## CRediT authorship contribution statement

**Daniel M. Callahan:** Writing – original draft, Visualization, Project administration, Methodology, Investigation, Conceptualization. **Hayes W. Miller:** Writing – original draft, Software, Investigation. **Ryan L. Frazier:** Writing – review & editing, Validation, Project administration. **E. Charles Osterberg:** Writing – review & editing, Validation, Supervision. **Aaron A. Laviana:** Writing – review & editing, Validation, Supervision, Project administration, Investigation, Conceptualization.

## Ethics

Written informed consent was obtained from the patient for publication of this case report and accompanying images. This study was conducted in accordance with institutional guidelines. Formal IRB approval was waived due to the retrospective nature of a single case report.

## Funding statement

This research did not receive any specific grant from funding agencies.

## Conflict of interest

The authors declare no conflicts of interest.
